# Urolithin A’s Antioxidative, Anti-Inflammatory, and Antiapoptotic Activities Mitigate Doxorubicin-Induced Liver Injury in Wistar Rats

**DOI:** 10.3390/biomedicines11041125

**Published:** 2023-04-07

**Authors:** Shahid Karim, Batoul Madani, Abdulhadi S. Burzangi, Mohammed Alsieni, Mohammed A. Bazuhair, Maha Jamal, Hussam Daghistani, Mohammed O. Barasheed, Huda Alkreathy, Mohammad Ahmed Khan, Lateef M. Khan

**Affiliations:** 1 Department of Pharmacology, Faculty of Medicine, King Abdulaziz University, Jeddah 21589, Saudi Arabia; 2 Department of Clinical Biochemistry, Faculty of Medicine, King Abdulaziz University, Jeddah 21589, Saudi Arabia; 3 Regenerative Medicine Unit, King Fahd Medical Research Center, King Abdulaziz University, Jeddah 21589, Saudi Arabia; 4 Department of Pathology, Faculty of Medicine, King Abdulaziz University, Jeddah 21589, Saudi Arabia; 5 Department of Pharmacology, School of Pharmaceutical Education and Research, Jamia Hamdard, New Delhi 110062, India

**Keywords:** chemotherapy, drug-induced liver injury, urolithin A, apoptosis, anti-inflammatory, oxidative stress

## Abstract

Human colon microbiota produce a metabolite called urolithin A (URO A) from ellagic acid and linked compounds, and this metabolite has been demonstrated to have antioxidant, anti-inflammatory, and antiapoptotic activities. The current work examines the various mechanisms through which URO A protects against doxorubicin (DOX)-induced liver injury in Wistar rats. In this experiment, Wistar rats were administered DOX intraperitoneally (20 mg kg^−1^) on day 7 while given URO A intraperitoneally (2.5 or 5 mg kg^−1^ d^−1^) for 14 days. The serum levels of aspartate aminotransferase (AST), alanine aminotransferase (ALT), and gamma glutamyl transferase (GGT) were measured. Hematoxylin and eosin (HE) staining was used to evaluate histopathological characteristics, and then antioxidant and anti-inflammatory properties were evaluated in tissue and serum, respectively. We also looked at how active caspase 3 and cytochrome c oxidase were in the liver. The findings demonstrated that supplementary URO A therapy clearly mitigated DOX-induced liver damage. The antioxidant enzymes SOD and CAT were elevated in the liver, and the levels of inflammatory cytokines, such as TNF-α, NF-kB, and IL-6, in the tissue were significantly attenuated, all of which complemented the beneficial effects of URO A in DOX-induced liver injury. In addition, URO A was able to alter the expression of caspase 3 and cytochrome c oxidase in the livers of rats that were subjected to DOX stress. These results showed that URO A reduced DOX-induced liver injury by reducing oxidative stress, inflammation, and apoptosis.

## 1. Introduction

Doxorubicin (DOX) is a broad-spectrum anthracycline antibiotic that is frequently used in conjunction with other medications to treat solid tumors, lymphomas, and leukemias [1]. The high toxicity of DOX to organs such as the liver, heart, kidneys, lungs, testes, and nervous system [2,3] limits its usage in clinical settings [2,3]. DOX has significant anticancer efficacy; nevertheless, it cannot be used since it has deleterious effects on cells that are not malignant [4]. DOX is an inhibitor of topoisomerase II, which causes apoptosis by oxidative damage to DNA caused by free radicals. The primary metabolic pathway of DOX metabolism involves oxidation mediated by CYPs, especially CYP3A4 (and CYP2D6, 2B6, and 1B1 to a lesser extent), while the secondary minor routes involve one-electron reduction and deglycosidation facilitated by oxidoreductases such as CYP-reductase (CPR), NADH- and NADPH-dehydrogenases, and xanthine dehydrogenase, which leads to the buildup of toxic and immunogenic intermediates such as Doxorubicinol, and other toxic metabolites that can harm the liver are produced [5,6]. Numerous studies have linked DOX therapy to a variety of histological alterations in liver tissues, including vacuolation of hepatocytes, localized necrosis, cellular edema, hyperplasia of the bile ducts, and lymphocyte infiltration [7]. Increased blood levels of alkaline phosphatase (ALP), aspartate transaminase (AST), and alanine transaminase (ALT), all of which point to liver injury and necrosis, are also linked to DOX treatment [8]. The hepatotoxicity of DOX is caused by the production of reactive oxygen species (ROS) during metabolism. Redox imbalance and oxidative stress are caused by these ROS, which deplete antioxidant enzymes [9]. DOX also increases the expression of apoptotic proteins, which is another factor to consider [10]. The increased production of many proinflammatory cytokines in liver tissues indicates that DOX also causes inflammation [11].

Urolithin A (URO A), also known as 3,8-dihydroxy-urolithin, is a kind of microflora human metabolite that is formed by the gut microbiota from ellagitannins and ellagic acid [12]. It has been found to have a high potential for scavenging free radicals [13]. Numerous in vivo studies have shown that URO A has a wide range of pharmacological properties, including antioxidant, anti-inflammatory, and antiapoptotic effects [14]. Urolithin A’s potent antioxidant activity suggests it might be helpful in warding off the pathological diseases brought on by oxidative stress [15]. A few studies show that URO A plays a vital role in regulating metabolism, insulin resistance, and obesity, all of which may aid in improving hepatic hemostasis, but there is still lack of clarity about how URO A protects the liver [16,17]. In addition, the results of the first clinical study of URO A in humans showed that it was biologically safe and enhanced mitochondrial activity in the aged human participants [18]. Therefore, it may be likely to prevent DOX hepatotoxicity by minimizing oxidative stress, apoptosis, and inflammation.

In addition, both colon cancer cell lines and an in vivo rat model have been used to investigate urolithin’s capacity to modulate the expression of phase I and phase II detoxifying enzymes [19]. Critical to the breakdown of chemical carcinogens, polycyclic aromatic hydrocarbons such as DOX are the Phase I and II enzymes [20]. Interestingly, the polycyclic aromatic hydrocarbons and other environmental toxicants are converted into a more polar and water-soluble metabolite by the phase I and phase II enzymes, which is then eliminated in the bile or urine [21]. URO A increased CYP1A1 and UGT1A10 expression and activity in colon cancer [19,22].

DOX is a crucial component of cancer chemotherapy; hence, many efforts have been made to reduce its side effects. These include the use of analogues and combination treatments, as well as dose optimization. However, no conclusive results have been achieved as of yet [23]. Natural products are, therefore, seen as a promising strategy for addressing such issues [24]. Therefore, the goal of this investigation is to examine the possible protective action of URO A against DOX-induced hepatotoxicity in Wistar rats.

## 2. Methods

### 2.1. Chemicals

Urolithin A (S5312) was purchased from Selleckchem (Selleck Chemicals LLC., Houston, TX, USA). Doxorubicin (DOX) (Fersenius Kabi AG, Germany) and other chemicals were of the finest grade. Urolithin A was dissolved in 5% DMSO at a concentration of 20 mgmL^−1^ and stored at 4 °C in the dark. Prior to injection, a URO A aliquot of the stock solution was adjusted in PBS at room temperature.

### 2.2. Animals

To conduct this study, 24 male Wistar rats (weighing between 200 and 230 g) were purchased from the animal facility of the Faculty of Pharmacy at King Abdulaziz University. The animals had access to water and food 24/7, were housed in a climate-controlled environment with a temperature range of 22–25 °C and humidity at approximately 60%, and were exposed to a light/dark cycle of 12 h each day. The protocols for animal care and experimentation were approved by the Research Ethics Committee of the Faculty of Pharmacy at King Abdulaziz University (Approval date: 5 September 2021; Approval Reference #PH-1443-07). All animal-related work, such as euthanasia and the collection of blood and tissues, were conducted as per the international norms, with special emphasis on its humane nature.

### 2.3. Experimental Design

**Figure 1:**
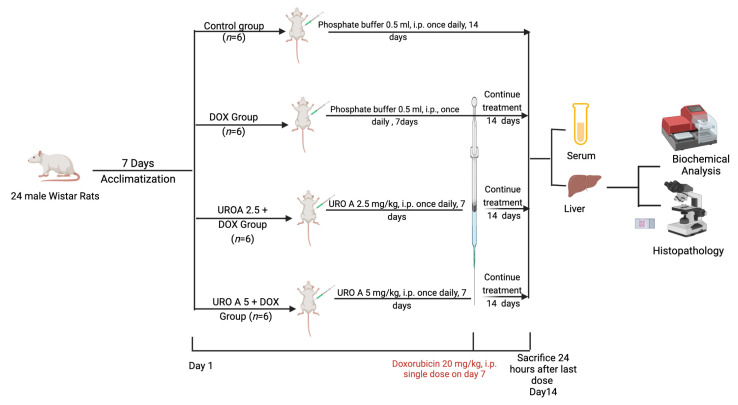
Experimental design of the proposed work.

Wistar rats were divided randomly into four groups (n = 6): a control group, a DOX group, a URO A at 2.5 mgkg^−1^ with DOX group, and a URO A at 5 mgkg^−1^ with DOX group (Figure 1). URO A doses were based on an earlier study [25].

Briefly, rats were acclimatized for seven days in animal housing. The control and DOX groups received PBS consecutively for 14 days at a dose of 0.5 mL i.p. once daily. The URO A treatment groups received intraperitoneal URO A in 5% DMSO in PBS, and a single dose of DOX (20 mgkg^−1^, i.p.) [26] was administered 60 min after URO A administration on the 7th day before continuing with assigned doses for 7 consecutive days.

Ketamine (50 mgkg^−1^) and xylazine (5 mgkg^−1^) via I.P. were used to induce aesthesia in rats 24 h after the last dosage of allocated treatment. Serum was extracted by drawing blood from the retro-orbital plexus, letting it clot for 15 min, and then centrifuging it at 3000 RPM for 10 min at 4 °C. After decapitating the rats, the livers were removed immediately and washed carefully in ice-cold saline. Then, Whatman filter paper was used for drying the excised livers. A 10% neutral formalin solution was used to preserve liver sections for further histopathological analysis. The remaining portions were flash-frozen in liquid nitrogen and stored at −80 °C with the sera until further analysis was possible.

### 2.4. Assessment of Hepatic Function Serum Markers

Alanine aminotransferase (ALT), aspartate aminotransferase (AST), lactate dehydrogenase (LDH), and gamma glutamyl transferase (GGT) activities in the serum were assessed with colorimetric ELISA kits (MBS269614, MBS264975, MBS269777, and MBS9343646, respectively; Mybiosource, San Diego, CA, USA).

### 2.5. Histopathological Examination

Post euthanization, under aseptic conditions, tissues from the rat liver were fixed by putting them in 10% neutral formalin and then embedding them in paraffin. The tissues were then cut into 5 µm thick slices and stained with hematoxylin and eosin (H&E). The sections were then photographed with an Olympus light microscope (model BX51TF, made in Japan). This histopathological assessment was conducted by an expert pathologist who was blinded about the assigned treatment groups. Hepatic damage was semi-quantitively assessed in a low-power field depending on the severity and tissue damage percentage, as stated before [27]. A grading system (0–4) was utilized, where 0 was normal tissue, 1 was <25% of tissue injured, 2 was 26–50% of tissue damaged, 3 was tissue damage in 51–75%, and 4 was tissue damage >75%. The pathological criteria employed for evaluation were liver lobule architecture, vascular congestion, hepatocyte necrosis, and inflammatory cells.

### 2.6. Assessment of Oxidative Status

According to Graham’s protocol, liver tissues were homogenized in a ten-fold volume of ice-cold PBS [28]. This was followed by collection of the supernatant, which was used for oxidative stress analysis. Mybiosource ELISA kits were utilized to assess the hepatic content of malondialdehyde (rat MDA; MBS738685, Mybiosource, San Diego, CA, USA) and the enzyme activities of superoxide dismutase (SOD; Cat. No. MBS036924, Mybiosource, San Diego, CA, USA) and catalase (CAT; Cat. No MBS726781, Mybiosource, San Diego, CA, USA). The tissue parameters were normalized to mg of protein.

### 2.7. Assessment of Inflammatory Markers

TNF-α (Cat. No. MBS2507393), NF-kB (Cat. No. MBS268833), and IL-6 (Cat. No. MBS726707) inflammatory markers were assessed in liver homogenate using ELISA kits according to the manufacturer’s instructions. MyBioSource, Inc. manufactured all the kits (SanDiego, CA, USA). The tissue parameters were normalized to mg of protein.

### 2.8. Assessment of Apoptosis

Caspase 3 (Casp3) and cytochrome c oxidase (CcO) (MBS018987 and MBS700786, respectively; Mybiosource, San Diego, CA, USA) were assayed in the liver tissue homogenate for the apoptosis markers mentioned above. The tissue parameters were normalized to mg of protein.

### 2.9. Statistical Analysis

All results are shown as mean ± SEM. One-way ANOVA followed by Holm–Šídák’s multiple comparisons tests were performed using GraphPad Prism v 9.4.0, San Diego, CA, USA. In all cases, *p* < 0.05 was considered statistically significant.

## 3. Results

### 3.1. Urolithin A Effect on Liver Function

**Figure 2:**
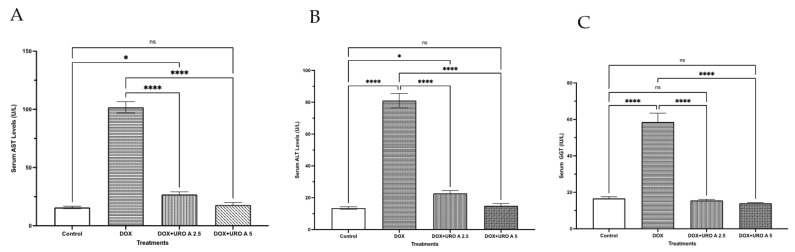
Effect of URO A on serum biomarkers of hepatotoxicity induced by DOX in rats: (**A**) AST activity, (**B**) ALT activity, and (**C**) GGT activity. Data are presented as mean ± SEM (n = 6); * indicates significantly different at *p* < 0.05 and **** at *p* < 0.0001; ns: nonsignificant.

The first series of tests examined the protective effects of URO A (Figure 2) at two different dosages against DOX-induced hepatotoxicity in rats. DOX treatment dramatically elevated serum AST activity by 547.5 percent, as seen in Figure 2A. At 2.5 mgkg^−1^ and 5 mgkg^−1^, URO A substantially reduced the rise in AST activity by 73.5 and 82.4 percent, respectively. Treatment with DOX alone increased blood ALT levels by 498.9 percent compared with the control group. Nonetheless, previous treatment with URO A at the two used dosages curtailed the rise in serum ALT activity caused by DOX by approximately 71.9 and 81.1 percent, respectively (Figure 2B). The serum GGT followed the same trend as seen in Figure 2C. At 2.5 mgkg^−1^ and 5 mgkg^−1^, URO A substantially decreased DOX-induced serum GGT activity by 73.5 and 76.1 percent, respectively.

### 3.2. Histopathological Changes after URO A Treatment

**Figure 3:**
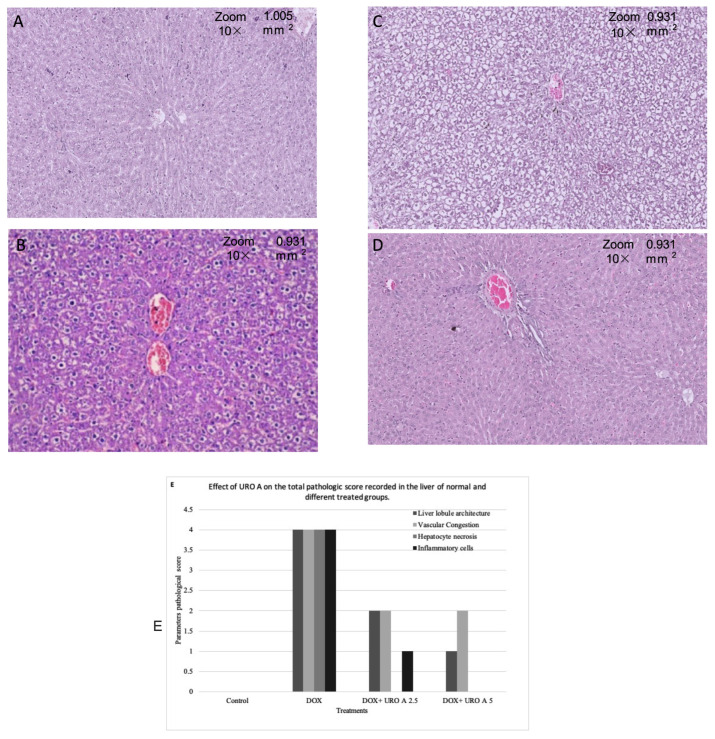
Representative histopathological examination of hematoxylin-and-eosin-stained photomicrographs of rat livers: (**A**) Normal liver histology showing no evidence of necrosis or inflammatory cell infiltration. (**B**) DOX-treated group with intralobular mononuclear inflammatory infiltrations, congestion of pericentral vessels and sinuses, and degeneration of hepatocytes increasing vacuolation in the cytoplasm of hepatocytes and appearing as indistinct, clear vacuoles. (**C**) DOX with URO A (2.5 mgkg^−1^) treatment group exhibiting considerable improvement in necrosis, inflammatory cells, and glycogen infiltration. (**D**) DOX with URO A (5 mgkg^−1^) treatment group showing marked improvement in vacuolization and inflammatory cells with moderate improvement in necrosis extending from the central zone to the midzone. (**E**) Semiquantitative pathologic score depending on the severity and tissue damage.

Microscopically, the liver tissues of control animals exhibited normal histology with a typical liver histological appearance and lacked necrosis or inflammatory cells (Figure 3A). In rats treated with DOX alone, significant hepatic injury was seen. Intralobular mononuclear inflammatory infiltrations and Mallory bodies resulting from hepatocyte degeneration increased vacuolation appeared as indistinct, clear vacuoles, indicating glycogen infiltration and demonstrating necrosis extending from the central zone to the midzone (Figure 3B). Animals treated with URO A (2.5 mgkg^−1^) prior to DOX administration revealed moderate improvement in necrosis and inflammation, with alterations equivalent to those of the control group (Figure 3C). The DOX with URO A (5 mgkg^−1^) group had the greatest improvement in hepatic parenchyma. Several examined sections revealed what seemed to be normal hepatic architecture, infiltration of dispersed inflammatory cells, and no clear microscopic changes (Figure 3D).

### 3.3. URO A Treatment Attenuates Oxidative Stress

**Figure 4:**
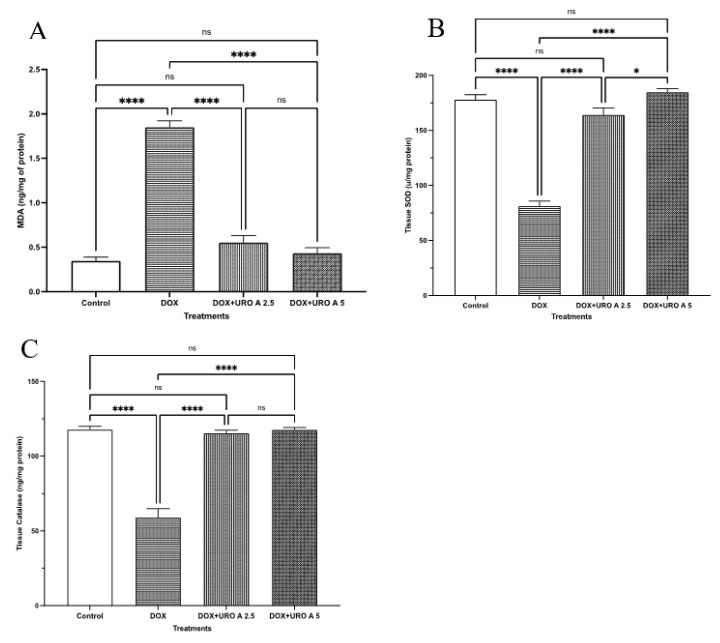
Effect of URO A on oxidative status in DOX-induced hepatotoxicity in rats: (**A**) effect on MDA levels, (**B**) effect on SOD activity, and (**C**) effect on catalase activity. Data are presented as mean ± SEM (6 rats per group); * *p* < 0.05; **** *p* < 0.001; ns: non-significant (one-way ANOVA followed by Holm–Šídák’s multiple comparisons test; *p* < 0.05).

In the subsequent series of tests, the protective effect of URO A on the oxidative state of rats with DOX-induced hepatotoxicity was determined. According to Figure 4A, DOX exposure elevated tissue MDA (overproduction of MDA induced by an increase in free radicals) by 434.68% of the control value. URO A decreased this DOX-induced rise by roughly 19.46% at a dosage of 5 mgkg^−1^, but the decrease at a dose of 2.5 mgkg^−1^ was minor. In terms of SOD content, the groups treated with URO A plus DOX contained considerably more SOD than the group treated with DOX alone. The SOD levels in the 2.5 mgkg^−1^ and 5 mgkg^−1^ UROA groups were 101.72% and 127.03% higher, respectively, than the DOX group. When comparing the SOD levels of the DOX group to those of the control group, a 54.26% decrease was found (Figure 4B). At dosages of 2.5 mgkg^−1^ and 5 mgkg^−1^ of URO A, hepatic catalase activity was 95.36 and 98.91 percentage points greater than that in the DOX group, respectively. Rats treated with DOX exhibited a 49.81% decrease in catalase activity relative to control rats (Figure 4C).

### 3.4. URO A Treatment Modulates Hepatic Inflammation

The potential of URO A as an anti-inflammatory agent was assessed in stressed liver tissues. The top row of Table 1 shows that co-administration of URO A at 2.5 mgkg^−1^ significantly reduced the TNF-α level by 26.76% and, for 5 mgkg^−1^, by 47.07% compared with the DOX group. The middle row of Table 1 shows that IL-6 was markedly increased by 170.02% with the administration of DOX compared with control group. However, URO A treatment significantly reversed this increase by 59.01% and 62.73% at 2.5 mgkg^−1^ and 5 mgkg^−1^, respectively. The bottom row of Table 1 shows that DOX exposure also caused a marked elevation of 304.72% in NF-κB levels, and this surge was significantly attenuated by 73.43% and 77.50% with the supplementary administration of URO A at 2.5 mgkg^−1^ and 5 mgkg^−1^, respectively.

### 3.5. URO A Treatment Leads to Caspase 3 and Cytochrome C Oxidase as Markers of Apoptosis

**Figure 5:**
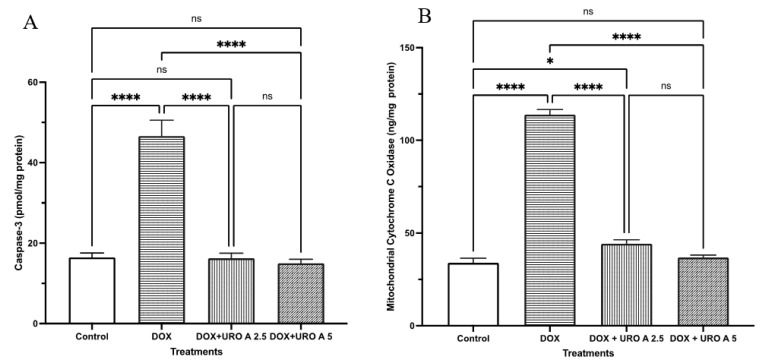
Effect of URO A on apoptotic markers in DOX-induced hepatotoxicity in rats: (**A**) effect on caspase 3 levels and (**B**) effect on mitochondrial cytochrome c oxidase. Data are presented as mean ± SEM (6 rats per group); * *p* < 0.05 and **** *p* < 0.001; ns: non-significant (one-way ANOVA followed by Holm–Šídák’s multiple comparisons test).

To gain further insight into the hepatic cytoprotective activity of URO A in DOX-stressed rodent livers, the expression of caspase 3 was assessed after the DOX and URO A interventions using ELISA assays. As can be seen in Figure 5A, the caspase 3 level was significantly increased in DOX-treated rat liver tissue by 183.11% versus control group. On the other hand, URO A treatment at the doses 2.5 mgkg^−1^ and 5 mgkg^−1^ significantly attenuated the increase in caspase 3 levels, as it declined these values by 65.15% and 67.85% versus the DOX group, respectively.

We also compared the expression of cytochrome oxidase as an important marker of cellular damage leading to apoptosis. The expression of the cytochrome c oxidase was assessed after the DOX and URO A treatment using ELISA assays. We observed that the cytochrome c oxidase level was significantly increased in DOX-treated rat liver tissue by 183.11% compared with the control group (Figure 5B). On the other hand, URO A treatment at the doses 2.5 mgkg^−1^ and 5 mgkg^−1^ significantly attenuated the increase in caspase 3 levels, as it declined these values by 61.15% and 67.70% versus the DOX group, respectively, as shown in Figure 4B.

## 4. Discussion

Hepatic injury due to chemotherapy is diagnosed by keeping an eye on raised liver enzyme function tests, which can be difficult to do in a clinical setting. It has been estimated that between 30 and 40% of patients receiving DOX had hepatotoxicity, based on the findings of earlier reports [29,30]. Nevertheless, DOX may have unintended side effects on non-cancerous cells, restricting its use in therapeutic settings [1,2].

Acute toxicity caused by a single dosage of DOX in animals has been linked to oxidative damage [31,32]. Moreover, acute toxicity, subacute toxicity, and chronic toxicity have all been described for DOX [33,34]. Damage to the liver, kidneys, and heart can result after a single dosage of DOX (typically between 5 and 30 mg/kg) in the model acute toxicity. The use of several low doses of DOX over a period of 2–12 weeks is required to generate the chronic toxicity of DOX [6,35]. Thus, we followed the lead of other investigations and employed a single dosage of DOX to cause acute liver injury in the present investigation. In addition, the single dosage of DOX (20 mgkg^−1^) that we employed in the current study is equivalent to a high single dose in the clinic for treating cancer patients [26].

Acute hepatoxicity may be shown in elevated serum transaminases [26,36]. Transport mechanisms and membrane permeability are both affected by damage to the liver. This may cause the release of certain enzymes from liver cells, leading to abnormally low levels of aminotransferases (ALT, AST, and GGT). On the other hand, elevated levels of these enzymes in the blood might serve as clinical indicators of liver damage [9,30]. In this study, the serum concentrations of ALT, AST, and GGT all rose noticeably when DOX was administered. When the liver is damaged, hepatocytes, where ALT is stored, are more likely to leak out. Diagnosing liver damage in a clinical environment requires careful regulation of liver enzymes and inflammatory agents with aberrant enzymatic contents [37]. Yet, it has also been established that the degree of hepatic injury is correlated with a rise in both AST and ALT activity [9]. A strong protective effect against hepatotoxicity was seen after supplementation with URO A (2.5 and 5 mgkg^−1^). This was due to a marked reduction in enzymatic activity. These results showed that the serum AST, ALT, and GGT were elevated after DOX treatment as compared with control group. Similar results were seen in a DOX-induced hepatic damage animal model [26].

For decades, DOX-induced organ toxicity has been associated with oxidative stress [9]. Oxidative stress induces lipid peroxidation in the cell membrane, the impairment of intracellular vital components such as proteins and deoxyribonucleic acid, the induction of mitochondrial dysfunction, and the activation of apoptosis-related proteolytic cascades, eventually leading to catastrophic injury and cell death [38,39]. DOX-induced hepatic damage, as evidenced by the elevated levels of liver enzymes and histopathological changes, was linked to oxidative stress in the current study. Nonetheless, URO A co-administration dramatically alleviated DOX-induced oxidative stress, as indicated by decreased lipid peroxidation and enhanced antioxidant enzyme activities in terms of MDA depletion and SOD and CAT activities, respectively. Recent research found that administering URO A resulted in a considerable reduction in oxidative stress indicators and liver histological characteristics in aging mice with DOX-induced liver injury [40]. The cytoprotective effects of URO A may be explained by the fact that it improves cell viability, reduces ROS generation, and boosts the physiological activity of various antioxidant defense mechanisms, such as CAT and SOD, as demonstrated by Cásedas et al. [31].

Increased ROS and RNS, as well as endogenous antioxidant depletion, cause a large immunological response [32]. Inflammation is one such complicated process caused by the activation of immune cells [41]. Many studies have shown increased inflammatory mediators, such as the infiltration of inflammatory cells, indicating that inflammation plays a role in DOX-linked organ damage [42,43]. TNF-α is another important marker in the pro-inflammatory cytokine–chemokine network induced by DOX [44,45]. Interleukin-6 (IL-6) is a key cytokine in hepatic inflammatory response, similar to TNF-α [46,47].

The observed hepatoprotective benefits in this study might potentially be mediated by URO A’s anti-inflammatory action, since we observed lower TNF- α and IL-6 production, both of which are major inflammatory cytokines. The current findings support earlier works emphasizing URO A’s powerful anti-inflammatory effect, as seen by reduced IL-6 and TNF-α production from peripheral blood mononuclear cells [40]. Involvement of the nuclear transcription factor NF-κB in the pathophysiology of drug-induced hepatotoxicity is well established [48]. It is more able to form complexes with its inhibitors, IKα and IKβ, when it is in its inactive state. Activation of IKα or IKβ in response to an insult or oxidative stress enhances the phosphorylation of IKβ, which in turn releases NF-κB and facilitates its translocation into the nucleus. These findings are also consistent with URO A’s known anti-inflammatory properties, since it inhibits NF-kB activation and the generation of proinflammatory cytokines [49,50,51].

Mitochondria are essential for intracellular energy metabolism. Protein, lipids, and DNA in the mitochondria are altered by oxidative stress [18,52,53]. Destruction of the mitochondrial structure causes abnormalities in mitochondrial function, which are linked to apoptosis and caspase activation, resulting in hepatic cell damage [46]. ROS-induced damage to mitochondrial structures such as membranes leads to the opening of mitochondrial permeability transition pores (MPTPs), resulting in the release of mitochondrial cytochrome c in cytosol. This triggers downstream events, such as the activation of caspase 9 and caspase 3, resulting in DNA damage and the impairment of mitochondrial function [39,53,54,55,56]. As a result, mitochondrial dysfunction is important in the pathogenesis of DOX-induced hepatotoxicity [34,52].

The mechanism of URO A’s protection against hepatic cell apoptosis is the suppression of apoptosis via the control of mitochondrial apoptotic pathways. We verified increases in cytochrome c oxidase and caspase 3 levels following DOX-induced hepatotoxicity in the current study using ELISA-based assays. Our findings demonstrated that URO A intervention dramatically decreased both caspase 3 and cytochrome c oxidase levels, as reported by Kim et al. [38]. However, the antiapoptotic impact of URO A described above may need to be further investigated and validated due to a lack of mRNA expression of other markers, such as BAX, BCL2, and p53 (a significant factor in apoptosis in mammals), which was a drawback of our investigation. As such, our data provide the first direct evidence of a hepatic protective effect of URO A in DOX-induced liver injury. As a result, it is possible to conclude that URO A can be used as a phytotherapeutic compound that protects against DOX-induced liver impairment.

Further research into the impact of URO A regulatory activity on the Sirt-1/FOXO1/NF-kB axis is needed, as Sirt-1 is also a critical player in DOX-induced toxicity. More research is needed to validate URO A’s safety and capacity to prevent DOX hepatotoxicity in the clinical context.

## Figures and Tables

**Table 1:** The effect of URO A on DOX-induced alterations in TNF-α, IL-6, and NFκB. Data are presented as mean ± SEM (6 rats per group); a: vs. control; b: vs. DOX (one-way ANOVA followed by Holm–Šídák’s multiple comparisons test; *p* < 0.05).

Treatment	Control	DOX	DOX with URO A at 2.5 mgkg^−1^	DOX with URO A at 5 mgkg^−1^
Inflammatory Marker				
TNF-α (pg/mg protein)	13.66 ± 0.53	24.60 ± 1.50 ^a^ (*p* < 0.0001)	12.56 ± 0.17 ^b^ (*p* < 0.0001)	13.02 ± 0.45 ^b^ (*p* < 0.0001)
IL-6 (pg/mg protein)	4.77 ± 0.30	12.88 ± 0.78 ^a^ (*p* < 0.0001)	5.28 ± 0.22 ^b^ (*p* < 0.0001)	4.8 ± 0.33 ^b^ (*p* < 0.0001)
NF- κB (ng/mg protein)	15.68 ± 0.6	63.46 ± 6.56 ^a^ (*p* < 0.0001)	16.86 ± 0.92 ^b^ (*p* < 0.0001)	14.28 ± 0.43 ^b^ (*p* < 0.0001)

## Data Availability

All data included in the main text.
